# Early vascular modifications after endoscopic endonasal pituitary surgery: The role of OCT-angiography

**DOI:** 10.1371/journal.pone.0241295

**Published:** 2020-10-29

**Authors:** Gilda Cennamo, Domenico Solari, Daniela Montorio, Maria Rosaria Scala, Antonietta Melenzane, Federica Fossataro, Teresa Somma, Fausto Tranfa, Luigi Maria Cavallo

**Affiliations:** 1 Public Health Department, University of Naples “Federico II”, Naples, Italy; 2 Division of Neurosurgery, Department of Neurosciences, Reproductive Sciences and Dentistry, University of Naples “Federico II”, Naples, Italy; 3 Eye Clinic, Department of Neurosciences, Reproductive Sciences and Dentistry, University of Naples “Federico II”, Naples, Italy; Bascom Palmer Eye Institute, UNITED STATES

## Abstract

**Purpose:**

The aim of the present study is to analyze the changes in retinal vessel density (VD), using Optical Coherence Tomography Angiography (OCT-A), in patients that received endoscopic endonasal approach for the removal of an intra-suprasellar pituitary adenoma compressing optic chiasm.

**Methods:**

We evaluated the VD in Superficial Capillary Plexus (SCP), Deep Capillary Plexus (DCP), Radial Peripapillary Capillary (RPC) and the Foveal Avascular Zone (FAZ) area in a series of fourteen patients (7 males, 7 females, mean age 56 ± 13 years), as compared to healthy controls. We also detected the structural Spectral Domain (SD)-OCT parameters: Ganglion Cell Complex (GCC), Retinal Nerve Fiber Layer (RNFL), visual field parameters (Mean Deviation, Pattern Standard Deviation) and Best Corrected Visual Acuity (BCVA). These measurements were performed prior than surgery and 48 hours after.

**Results:**

The patients showed a significant decrease in VD of the macular and papillary regions, a significant increase in FAZ area, a significant impairment in SD-OCT, VF parameters and BCVA respect to 14 eyes of 14 healthy controls (p<0.05), at pre-op evaluation. In patients group the VD in SCP, DCP and RPC increased after surgery respect to baseline but the difference turned to be out statistically significant only in RPC (p = 0.003). Also the BCVA (p = 0.040) and the Mean Deviation at visual field (p = 0.015) significantly improved after surgery. While there was a reduction in structural OCT parameters but it was statistically significant only in GCC (p = 0.039). A positive correlation was found between the preoperative VD of the RPC, Mean Deviation, BCVA and the postoperative Mean Deviation (r = 0.426 p = 0.027; r = 0.624 p = 0.001; r = 0.515 p = 0.006).

**Conclusion:**

OCT-A allows to detect the early changes occurring within 48 hours after surgery showing that the improvement in retinal vessel density could occur before the recovery of the structural OCT parameters and can be a positive predictive factor for the functional recovery.

## Introduction

Visual impairment due to optic chiasm or optic nerve compression is most commonly detected in patients harboring intra-suprasellar lesions, e.g. pituitary adenomas, Rathke cleft cysts, craniopharyngiomas, accounting for up to 10–15% of all intracranial tumors [1]. Trans-nasal surgery for the treatment of these conditions, allows an optical surgical resection along with remarkable vision improvements [2]. The comprehension of several anatomical and functional information, i.e. the assessment of the structural integrity of the anterior visual pathway and the evaluation of visual loss along with definition of signs are crucial [3]. Elements associated with visual outcomes following the chiasm decompression surgery have been widely investigated: visual prognosis depends on the interval of compression, nature, and size of the lesion, patients’ age, visual fields, and visual acuity at the time of surgery and the presence of optic disc atrophy [4,5]. However, none of the aforementioned factors can allow the surgeon to define the specific postoperative course. On one side, visual field examination provides chiasmal status but it is not possible to quantify the impairment of lost or dysfunctional ganglion cells that can cause visual field alterations. Since the introduction of Optical Coherence Tomography (OCT), an imaging technique that utilizes near-infrared light interferometry to generate *in vivo* cross-sectional and three-dimensional volumes of retinal layers, it has been possible to reveal permanent structural anatomical changes, more precisely define the likelihood of functional recovery [6]. Recently, several optical coherence tomography angiography (OCT-A) methods have been developed especially for the reconstruction of three-dimensional noninvasive chorioretinal vascular imaging and are gaining popularity for the diagnosis of optic neuropathies [7–13].

To the best of our knowledge, only one study investigated the potential role of OCT-A in the measurement of vessel density to predict the postoperative visual recovery in chiasm compression due to pituitary tumors evaluating patients before and at 3 months after surgery [14]. The aim of our study is focused on the identification of early changes occurring in retinal vessel density (VD), using Optical Coherence Tomography Angiography (OCT-A), in patients that received Endoscopic Endonasal Approach (EEA) for the removal of an intra-suprasellar pituitary adenoma compressing the optic chiasm and to evaluate its eventual predictive factor of the functional recovery.

## Materials and methods

### Population and study design

In this study we enrolled 14 patients (twenty-eighty eyes), who underwent endoscopic endonasal approach (EEA) for the removal of intra-suprasellar pituitary adenoma compressing the optic chiasm, at the Division of Neurosurgery of the University of Naples “Federico II” from January to March 2019.

The inclusion criteria were: evidence of lesion compressing the chiasm at magnetic resonance imaging (MRI), the preoperative visual field (VF) impairment and absence of previous endoscopic endonasal surgery.

Exclusion criteria were: previous treatment for lesions compressing the chiasm, previous ocular surgery, congenital eye disease; high myopia (>6 diopters); diagnosis of glaucoma; any optic disc anomaly and macula disease; low-quality OCT and OCT-A images.

14 healthy subjects (8 females, 6 males, mean age 55 ± 10 years) with no optic disease were studied and served as control group for quantity analysis.

Each patients underwent pre-operative and post-operative (48 hours after) ophthalmological assessment, including: the measurement of best-corrected visual acuity (BCVA) according to the Early Treatment of Diabetic Retinopathy Study (ETDRS) [15] (the BCVA was converted into logarithm of the Minimum Angle of Resolution (log MAR) scale for statistical calculations), slit-lamp biomicroscopy, fundus examination, automatic perimetry test (Humphrey Field Analyzer using the 30–2 SITA-Standard algorithm of the Humphrey perimeter; Carl Zeiss Meditec, Dublin, CA, USA), evaluation of the structural Spectral Domain (SD)-OCT parameters (Ganglion Cell Complex and Retinal Nerve Fiber Layer) and OCT-A.

The study was approved by the Institutional Review Board of the University of Naples “Federico II” (clinical trials: NCT04425954) and all investigations adhered to the tenets of the Declaration of Helsinki. Written informed consents were obtained from the patients enrolled in the study.

### OCT angiography

OCT-A images with the Optovue Angiovue System (software ReVue XR version 2017.1.0.151, Optovue Inc., Fremont, CA, USA) that is based on split-spectrum amplitude de-correlation algorithm (SSADA) which uses blood flow as intrinsic contrast [16,17].

The software (AngioAnalytic^TM^) automatically calculated the vessel density (VD) of different retinal vascular networks in macular area over a 6 x 6 mm scan centered on the fovea and divided, according to the ETDRS classification of diabetic retinopathy, in whole image, fovea and parafovea. The retinal vascular networks analyzed were: the Superficial Capillary Plexus (SCP) and the Deep Capillary Plexus (DCP) [18].

The Angio Vue disc mode automatically segmented the Radial Peripapillary Capillary (RPC) analyzing the whole papillary region with an area scan of 4.5 x 4.5 mm. The VD was analyzed in the superficial retinal layers and extended from the ILM to the RNFL posterior boundary [19].

The vessel density (VD) was defined as the percentage area occupied by vessels in the analyzed region [20]. Angiovue software automatically calculated the Foveal Avascular Zone (FAZ) area in square millimetres over the 6 mm x 6 mm macular area in the full retinal plexus [21].

From the analysis were excluded the images with a signal strength index less than 40 and residual motion artefacts and incorrect segmentation.

### Surgical technique

Surgical treatment consisted of an endoscopic endonasal “standard” approach in all the cases, according to the techniques already described in previous publications [22–27]. All the procedures were performed using a rigid 0-degree endoscope, 18 cm in length and 4 mm in diameter (Karl Storz Endoscopy, Tuttlingen, Germany), as the sole visualizing tool.

### Statistical analysis

Statistical analysis was performed with the Statistical Package for Social Sciences (Version 20.0 for Windows; SPSS Inc, Chicago, Ill, USA). The unpaired Student’s t-test for independent samples was used to compare SD-OCT, OCT-A, visual field parameter and BCVA between patient before the surgery and control group. Moreover these parameters were analyzed in patients at baseline and 48 hours after the surgery by the paired Student’s t-test. Pearson’s correlation was assessed to evaluate the correlation between preoperative SD-OCT, OCT-A, visual field (VF) parameters, BCVA and postoperative Mean Deviation (MD). A p value of < 0.05 was considered statistically significant.

## Results

A total of twenty-eight eyes of fourteen patients (7 males, 7 females, mean age 56 ± 13 years) with a optic chiasm compression syndrome due to an intra and suprasellar lesion were enrolled in this study.

There were no statistically significant differences in terms of age (p = 0.747) and sex (p = 0.987) between the patients group and controls.

At SD-OCT exam, the Ganglion Cell Complex (GCC) and the Retinal Nerve Fiber Layer (RNFL) were significantly thinner in patients prior surgery respect to controls (95.78 ± 10.75 μm vs 102.32 ± 5.98 μm, p = 0.021; 95.25 ± 8.98 μm vs 103.82 ± 7.99 μm, p<0.001; respectively).

Also the VD of the SCP, DCP in all macular sectors and the VD of the RPC were significantly decreased (p<0.05), while the FAZ area was significantly increased (p<0.001) compared to healthy subjects.

Regarding the visual acuity and the visual field parameters, the patients showed a significant impairment in preoperative BCVA (-0.01 ± 0.39 logMAR vs -0.25 ± 0.05 logMAR, p = 0.002), MD (-5.87 ± 7 dB vs -0.58 ± 1.18 dB, p<0.001) and Pattern Standard Deviation (PSD) (5.86 ± 4.8 dB vs 2.11 ± 0.45 dB, p<0.001) as compared group (Table 1).

**Table 1:** Differences in OCTA, SD-OCT, visual field parameters and BCVA between preoperative patients and controls.

	Patients	Controls	p value
**Superficial Capillary Plexus (%)**			
***Whole image***	48.20 ± 3.81	54.21 ± 3.09	<0.001
***Parafovea***	50.86 ± 6.06	53.96 ± 3.62	0.024
***Fovea***	18.44 ± 8.58	27.60 ± 4.81	<0.001
**Deep Capillary Plexus (%)**			
***Whole image***	51.54 ± 8	56.21 ± 4.78	0.011
***Parafovea***	56.01 ± 5.95	59.14 ± 3.43	0.019
***Fovea***	36.24 ± 10.04	44.96 ± 6.22	<0.001
**Foveal Avascular Zone (mm**^**2**^**)**	0.306 ± 0.12	0.188 ± 0.06	<0.001
**Radial Peripapillary Capillary (%) *Whole image***	46.86 ± 2.98	53.21 ± 3.44	<0.001
**Ganglion Cell Complex (μm)**			
***Average***	95.78 ± 10.75	102.32 ± 5.98	0.021
**Retinal Nerve Fiber Layer (μm)**			
***Average***	95.25 ± 8.98	103.82 ± 7.99	<0.001
**Mean Deviation (dB)**	-5.87 ± 7	-0.58 ± 1.18	<0.001
**Pattern Standar Deviation (dB)**	5.86 ± 4.8	2.11 ± 0.45	<0.001
**BCVA (logMAR)**	-0.01 ± 0.39	-0.25 ± 0.05	0.002

Data expressed as mean ± SD; dB: decibel; BCVA: Best Corrected Visual Acuity; logMAR: logarithm of the minimum angle of resolution.

The unpaired Student’s t-test, p<0.05.

When comparing data prior and 48 hours after surgery, a statistically significant improvement in BCVA (-0.01 ± 0.39 logMAR vs -0.14 ± 0.16 logMAR, p = 0.040) was observed.

The structural OCT parameters (GCC and RNFL thicknesses) were reduced after surgery respect to baseline but a statistically significant difference was found only in GCC (95.78 ± 10.75 μm vs 92.89 ± 6.60 μm, p = 0.039).

Conversely, at OCT-A examination, the VD in RPC significantly increased after surgery (46.86 ± 2.98% vs 48.13 ± 3.67%, p = 0.003), also the VD of the SCP and DCP in each macular sector were slightly increased after surgery, although not statistically significant (SCP whole image: 48.20 ± 3.81% vs 48.91± 3.45%; p = 0.348; DCP whole image: 51.54 ± 8% vs 52.06 ± 7%; p = 0.711). On the contrary, there were no significant alterations in FAZ area after surgery.

**Fig 1:**
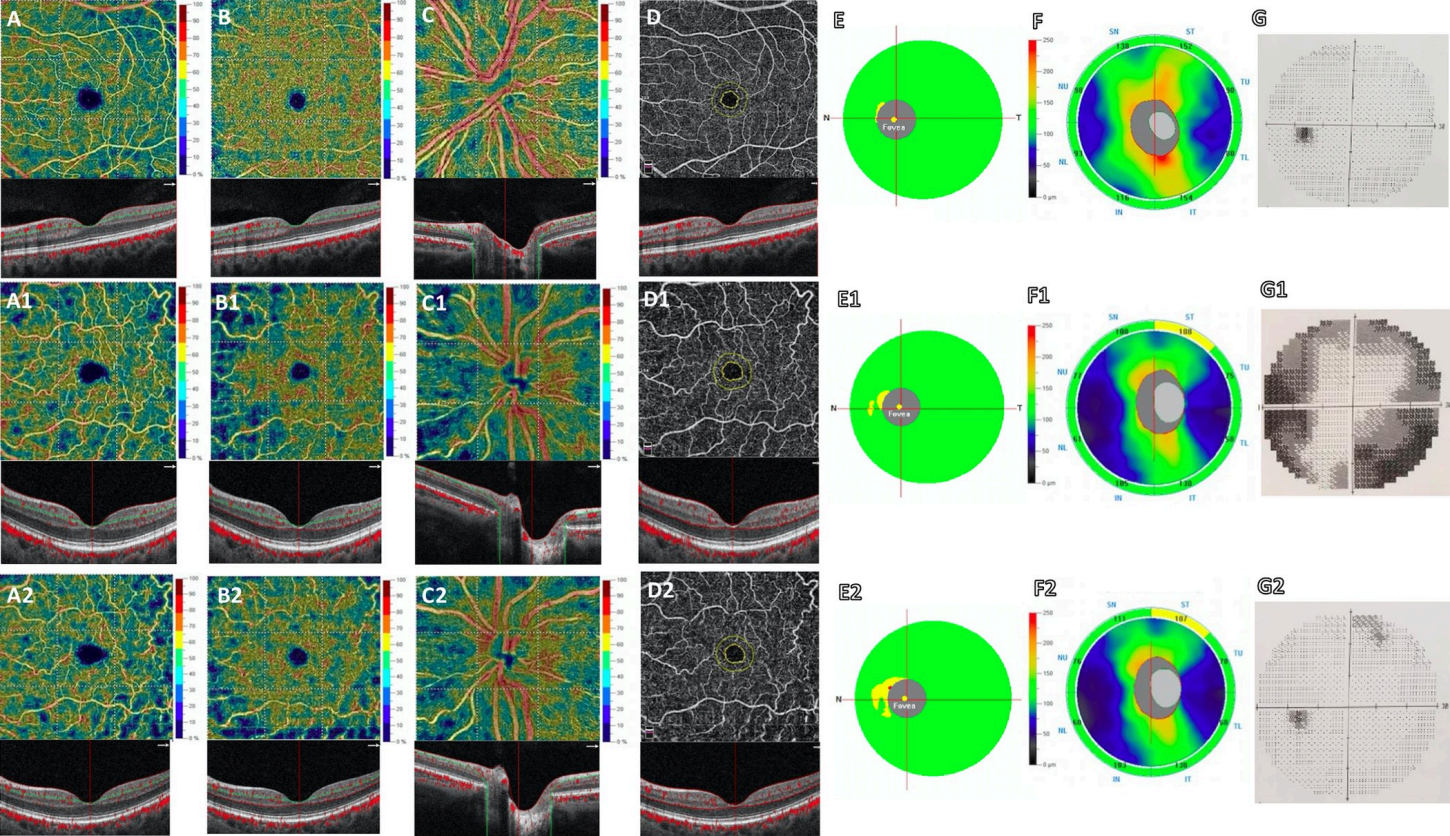
**Left eye of an healthy subject (45 years-old female) shows at Optical Coherence Tomography Angiography (OCTA) normal vessel density of the superficial capillary plexus (SCP) (A), deep capillary plexus (DCP) (B), radial peripapillary capillary (RPC) (C) and foveal avascular zone (FAZ) area (D).** Left eye of a patient (50 years-old female) with pitituary adenoma before surgery reveals a reduction of vessel density in SCP (A1), DCP (B1), RPC (C1) and an increase of FAZ area (D1) respect to healthy subject. No significant change in vessel density of the SCP (A2), DCP (B2), in FAZ area (D2) and an increased vessel density of the RCP (C2) are found in the same patient 48 hours after endoscopic endonasal surgery respect to baseline. Structural Spectral Domain OCT (SD-OCT) shows normal thickness of the Ganglion Cell Complex (GCC) (E) and Retinal Nerve Fiber Layer (RNFL) (F) in healthy control. The patient shows a focal reduction in GCC and RNFL (E1, F1) at baseline, a decrease in GCC (E2) and absence of changes in RNFL (F2) 48 hours after surgery. Respect to control (G), a significant impairment of visual field examination and a significant improvement are found at baseline (G1) and after surgery (G2), respectively.

Finally, concerning the visual field parameters, we noticed that the MD had significantly improved (-5.87 ± 7dB vs -3.19 ± 4.7dB, p = 0.015) while the PSD did not show any change after surgery (Table 2, Fig 1).

**Table 2:** Differences in OCTA, SD-OCT, visual field parameters and BCVA before and after surgery via endoscopic endonasal approach.

	Before	After	p value
**Superficial Capillary Plexus (%)**			
***Whole image***	48.20 ± 3.81	48.91 ± 3.45	0.348
***Parafovea***	50.86 ± 6.06	51.38 ± 5.20	0.642
***Fovea***	18.44 ± 8.58	19.23 ± 8.92	0.526
**Deep Capillary Plexus (%)**			
***Whole image***	51.54 ± 8	52.06 ± 7	0.711
***Parafovea***	56.01 ± 5.95	56.18 ± 5.77	0.887
***Fovea***	36.24 ± 10.04	37.42 ± 10.27	0.393
**Foveal Avascular Zone (mm**^**2**^**)**	0.306 ± 0.12	0.306 ± 0.13	0.994
**Radial Peripapillary Capillary (%) *Whole image***	46.86 ± 2.98	48.13 ± 3.67	0.003
**Ganglion Cell Complex (μm)**			
***Average***	95.78 ± 10.75	92.89 ± 6.60	0.039
**Retinal Nerve Fiber Layer (μm)**			
***Average***	95.25 ± 8.98	94.85 ± 10.46	0.561
**Mean Deviation (dB)**	-5.87 ± 7	-3.19 ± 4.7	0.015
**Pattern Standar Deviation (dB)**	5.86 ± 4.8	5.13 ± 4.75	0.319
**BCVA (logMAR)**	-0.01 ± 0.39	-0.14 ± 0.16	0.040

Data expressed as mean ± SD; dB: decibel; BCVA: Best Corrected Visual Acuity; logMAR: logarithm of the minimum angle of resolution.

The paired Student’s t-test, p<0.05.

In patients group the preoperative VD of the RPC (r = 0.426, p = 0.027), MD (r = 0.624, p = 0.001) and BCVA (r = 0.515, p = 0.006) were significantly correlated with the postoperative MD (Table 3).

**Table 3:** Correlations between preoperative SD-OCT, OCTA, visual field parameters, BCVA and postoperative MD.

	Postoperative MD	
	r	p
**Superficial Capillary Plexus**		
*Whole image*	0.016	0.938
*Parafovea*	-0.120	0.552
*Fovea*	0.432	0.124
**Deep Capillary Plexus**		
*Whole image*	-0.256	0.198
*Parafovea*	-0.214	0.285
*Fovea*	0.313	0.111
**Foveal Avascular Zone**	0.484	0.210
**Radial Peripapillary Capillary**		
*Whole image*	0.426	0.027
**Ganglion Cell Complex average**	0.430	0.125
**Retinal Nerve Fiber Layer average**	0.598	0.201
**Mean Deviation**	0.624	0.001
**Pattern Standard Deviation**	0.658	0.241
**BCVA**	0.515	0.006

Pearson’s correlation p<0.05.

## Discussion

In our study, we evaluated the preoperatory and postoperative Vessel Density in Superficial Capillary Plexus (SCP), Deep Capillary Plexus (DCP), Radial Peripapillary Capillary (RPC) and SD-OCT parameters (GCC and RNFL) 48 hours after endoscopic endonasal removal of an intra-suprasellar pituitary adenoma compressing the optic apparatus. The depth-resolved visualization of all the retinal vascular layers has been possible by the Optic Coherence Tomography Angiography (OCT-A), which is an innovative, non-invasive, and rapid imaging modality without the necessary cooperation of the patient [28,29]. OCT-A devices reflect the anatomical modifications using different algorithms, all of which based on the hypothesis that erythrocytes are the only moving particles within the blood vessels; hence, they can be detected as natural motion contrast. Since its recent introduction, OCT-A has gained popularity and has been applied to a broad spectrum of retinal disorders and other optic nerve diseases [28,30–37].

And yet few studies evaluated the role of OCT-A for definition of chiasm compression [38].

In our study, we explored the predictive role of OCT-A in visual recovery by investigating the early vessel density modifications that may occur 48 hours after endoscopic endonasal pituitary surgery.

The deformation of the optic chiasm caused by the growth of the pituitary adenomas is responsible for the occurrence of visual disturbances and, furthermore, for their entity. Considering the above, also in accordance with optic chiasm fibers disposition, the most common Visual Field defect is bitemporal hemianopsia, implying that the nasal retinal fibers are damaged [39,40]. Moreover, their major vulnerability may be attributed to their partial decussation before forming the optic tracts [41]: a significant positive relationship between the extent of bitemporal VF loss and the degree of central chiasmal elevation has been demonstrated [42]. Prieto et al. analyzed the correlation between optic chiasm distortions as quantified at MRI and their impact on the visual outcomes: the rate of visual loss was minor in patients with normal and/or compressed downward chiasms (less than 20%) and it was major in cases where chiasms were stretched from below [43]. These conditions were directly the final postoperative visual outcomes.

When the optic chiasm is directly compressed or its blood supply system is altered by the lesion, axonal injury and dysfunction or ganglion cell apoptosis may occur [44–46]. The role of OCT in the detection of anatomical changes during chiasmal compression has been widely investigated. It translates to a diminished thickness of both retinal nerve fiber layer (RNFL) and ganglion cell complex (GCC) [47], as stated in our previous publication, where patients with better preoperative values of p-RNFL and GCC presented a higher percentage of visual recovery [48]. Garcia et al. enrolled 34 patients with evidence of chiasmal compression and assessed the nasal RNFL thickness was an optimal prognostic factor for peripheral visual field recovery [4]. It is also well known there is a consistent correlation between the degree of visual function recovery and the degree of initial damage to retinal ganglion cell axons [44,45].

Different hypotheses have been postulated to relate the visual outcomes and timing of decompression through surgery. At first, the compression causes a physiological conduction block resulting in an impairment of anterograde and retrograde axoplasmic flow, creating a mainly reversible condition where an initial nerve suffering is established; if this condition persists, irreversible axonal damage will progress, as well as a visual dysfunction, leading to demyelination and advanced loss of ganglion cells. It may be associated with decreased retinal needs and may lead to capillary loss and that could be shown using optical coherence tomography angiography (OCT-A) [49].

Moon et al. studied the changes that occur after optic chiasmal decompression in terms of OCT parameters and visual field assessment and noticed that visual field recovery precedes the demonstrable retinal regeneration, albeit the established correlation between the anatomical and functional modifications [50]. It was thought the surgical timing may also play a role in terms of visual improvement.

Hence, we took into consideration the time elapsed between visual disturbances onset and surgical timing to assess any correlation with the visual recovery with regard to postoperative BVCA, VD, and Angio-OCT RPC value.

Accordingly, we first divided the patients into 3 groups; the first group received the surgical treatment within 30 days, the second one in a period between 45–60 days and, the third group in a period longer than 90 days. We initially compared their visual outcomes in order to identify a correlation and then, we calculated the presence of any difference among the groups between the preoperative and postoperative BVCA, VD and Angio-OCT RPC value assuming that those who received the surgical treatment before could score better visual improvements. Nonetheless, both correlations turned out to be not statistically significant. However, it cannot be underestimated that this can be related to the limited number of cases.

In agreement with previous studies, our preoperative values of VD of the SCP, DCP in all macular sectors, and the VD of the RPC were significantly decreased, along with RNFL and GCC, compared to the healthy subjects [14,49].

Up to date, only one study evaluated the potential role of OCT-A in the measurement of vessel density to predict the postoperative visual recovery in chiasm compression due to pituitary tumors. Lee et al. evaluated both OCT and OCT-A parameters of patients before and at a 3 months follow-up period after surgery. They did not find any statistical correlation between preoperative RNFL and GCC thickness and postoperative VF recovery, because of the great number presence of patients with mild VF but fewer structural anatomical changes, whereas they demonstrate a strong association between the average VD and the postoperative VF outcomes [14].

In the present study, we presumed that the early modification in VD at the OCT-A examination may precede the OCT parameters normalization and, consequentially, provide new instruments to predict the visual outcome of our patients. We assumed that during chiasmal compression there is initially a mechanical lower retinal perfusion along with reduced metabolic demand due to the optic injury, made by the lesion and we alleged that the early examination of the eventual normalization of VD could provide a prognostic factor on visual outcomes compared to the current ones such as RNFL or GCC.

Our findings demonstrate that VD of the SCP, DCP in each macular sector and RPC was increased after surgery even if the difference turned out to be statistically significant only in RPC (46.86 ± 2.98% vs 48.13 ± 3.67%, p = 0.003), notwithstanding the structural OCT parameters (GCC and RNFL thicknesses) were found ameliorated after surgery respect to baseline. Moreover, in patients group the postoperative MD was significantly correlated with VD of the RPC (r = 0.426, p = 0.027), MD (r = 0.624, p = 0.001) and BCVA which has shown better scores for all the patients (r = 0.515, p = 0.006) (Table 3).

According to our results, it might be said that the normalization of this OCT-A parameter after the chiasm decompression is strictly related to the peculiar RPC vessels anatomical location [51–54]. Michaelson has originally described them as a unique plexus of capillary bed with a limited distribution in the posterior pole and with a parallel orientation to the retinal nerve fiber layer axons [55]. Being the most superficial layer of capillaries lying in the inner part of the RNFL they might return to their original status thanks to the mechanical decompression effect achieved with the surgery [56–58]. Moreover, according to Yu et al., there is an established correlation between RPC distribution and RNFL thickness and their function is most likely to nourish its inner portion, as it is also postulated in other several publications regarding the physiopathology relative to ophthalmologic disease [51,52,59–61]. In agreement with previous observations, the RPC are the most prominent in the arcuate RNFL regions, and the positive correlation between RNFL thickness and RPC volume indicates a necessary supportive role of the RPCs in the RNFL [58].

Therefore, our data suggest that an early regularization of the RPC parameter alone may be considered as a sign of an optimal decompression achieved; this software can represent a non-invasive, rapid, and valid examination providing an accurate perspective of the functional and anatomical optic apparatus. At the same time, it can give information to both the neurosurgeon and the ophthalmologist in regards to the restitutio in integrum of the blood flow in the RNFL nourished by the RPC vessels, and provide a good prediction of visual recovery.

## Conclusions

Endoscopic endonasal surgery represents a sound and effective strategy for pituitary tumors removal. This technique allows a significant visual improvement in the majority of cases presenting with chiasm compression. OCT-A is a non-invasive innovative tool that allows an easy and fast vascular and anatomical retinal examination; consequentially, thus being a valid tool to estimate functional recovery. Although several factors should be taken into consideration, we retain that the early postoperative detection of the RPC parameter can be claimed as a reliable predicting factor of visual recovery in those patients presenting chiasm compression at onset. More extensive series and further efforts are required to define better the diagnostic and prognostic role of OCT-A along with its correlation with functional assays.
